# The Conversion of Pail Closets
*A paper read before the Royal Sanitary Institute at Blackburn.


**Published:** 1922-08

**Authors:** A. T. Gooseman

**Affiliations:** (Borough and Water Engineer, Blackburn)


### THE CONVERSION OF PAIL CLOSETS.*

**By A. T. GOOSEMAN (Borough and Water Engineer, Blackburn).**

VARIOUS devices have been tried from time to time for removing decomposing matter. All the elements known to the ancients have been laid under tribute—fire, air, earth and water; but to-day it is generally recognised that the water carriage system is the most effective for the speedy removal of matter liable to decompose, the storage of which, near our doors, even for a brief period, may have dangerous consequences. This system was in vogue centuries ago. The ruins of Pompeii show that latrines existed which were supplied with water, and from which the drains conveyed away the polluting matter. In this country prior to 1815, it was illegal to pass fæcal matter into sewers. Such matter was usually accumulated in cesspools. It was not until the year 1847 that the first Act of Parliament was passed which made it compulsory to pass fæcal matter into sewers.

In Lancashire we have some of the most progressive municipalities in the kingdom, who have provided their communities with an excellent water supply, sewage disposal works, public baths, public buildings, refuse destructors, hospitals, &c., but many towns have still in existence thousands of "fly factories" in the privy and pail closet. Sanitary laws are intended to give power to communities which single individuals cannot possess, and in this country these laws—to some extent—are permissive, so that we see in some districts the law is rigidly enforced, while community after community is still found living in the most insanitary conditions. It is over 50 years since that sanitary abomination known as the privy midden was practically abolished, and pails substituted in Blackburn. The progress of those days is partly responsible for the delay in converting the existing pails to the water carriage system. Two years ago I had a census taken of the closets in Blackburn, and the numbers were as follows :—

| | |
|---|---|
| Pail closets ... ... ... ... ... ... | 9,278 |
| Slop water closets ... ... ... ... ... | 2,541 |
| Privies... ... ... ... ... ... ... | 81 |
| Fresh water closets ... ... ... ... ... | 26,457 |
| TOTAL ... ... ... ... ... ... | 38,357 |

Over 20 years ago the Blackburn Corporation obtained borrowing powers under an Act of Parliament for the conversion of pails, privies, and slop-water closets into water closets. Under this Act the Corporation also obtained powers to have conversions carried out other than privy middens and cesspools, and at the same time to pay half the cost, providing the Corporation had not previously required alterations to be made in the closet accommodation of the premises concerned. In the case of any such previous alterations, if the owner could prove that these were carried out at the Corporation's request, the expense of any further alteration would be borne by the Corporation. Up to the beginning of last year the method adopted by the Corporation, in order to have these conversions carried out, was by agreement with the property-owners, the Corporation contributing a certain part of the cost. At that time it was £3 15s. for each conversion of a pail or slop water closet to the W.C. system, but nothing was allowed by the Corporation for the conversion of a privy midden. The average number of conversions carried out per year was only 50. The Corporation were dissatisfied with this rate of progress, and decided unanimously to prepare a scheme for the wholesale conversion of pails to the water carriage system. There were certain objections at the outset, but on going into these it was found the obstacles were imaginary ones.

It was suggested that our water supply was not sufficient to deal with the increased number of W.C.'s, but it was found by experiment in Blackburn that one conversion will only increase the consumption of water in the town by 15 gallons per day, and therefore 10,000 conversions would mean only an increased consumption of 150,000 gallons per day, which, in view of the fact that our average daily consumption of water is 4,000,000 gallons, would be a mere bagatelle. It was also contended that our sewers were not large enough to deal with the extra quantity of sewage when the conversions were completed, but as our dry-weather flow of sewage is 4,000,000 gallons per day, and it is necessary to carry in our sewers at least six times the dry-weather flow, *i.e.*, 24,000,000, the increase of 154,000 gallons of sewage, which the conversion scheme was found by experiment to entail, would be so small in comparison with the bulk that it could be ignored. In addition to this the practice in Blackburn during many months in the year has been to deposit the whole of the con-

* A paper read before the Royal Sanitary Institute at Blackburn.

tents of the pails into the sewers at two places, and therefore there was less objection to its being spread over the whole of the sewers in the borough.

At the present rate of wages it would cost the Corporation £7,759 per annum to collect and dispose of the pail contents which existed twelve months ago. When the whole of the conversions are completed it will probably have cost the Corporation in grants £60,000. They have borrowing powers for 25 years for this work. The interest and sinking fund of this amount at the present rate of interest is £4,444 per annum, and therefore, after allowing for a few pails on the outskirts of the town which cannot be converted—and will cost £150 per annum to collect—the scheme will show a saving of £3,000 per annum to the ratepayers, so there is a good margin to work on to allow for any fall in prices. Furthermore, after 25 years the interest and sinking fund on this amount will have been exhausted, and from that time onwards this scheme will entail no further expenditure from the rates, although the benefit of the financial saving and other advantages will still be enjoyed by the ratepayers. In addition, if the conversion of a pail closet costs the property-owner, say, £6, the substitution of a W.C. for a pail surely enhances the value of the property by more than this amount, and my experience is that in the majority of cases the tenant is only too pleased to pay a few coppers a week extra in rent, which will more than repay the landlord on his outlay, in order to live under more sanitary conditions.

The first step the Corporation took to abolish the pail system was to meet the property-owners, and after several interviews it was arranged to offer £8 to the owner who converted a pail to the W.C. system. At the same time it was decided to engage a staff of men under me to carry out such conversions as were not completed by the owner within one month after the notice to convert had been served, which incidentally enabled the Corporation to know the exact cost of the conversions. After a short time it was found that £8 was considerably more than half the cost of the work, as the average total cost of the first batch converted by direct labour was only £12 11s. This was partly due to the decrease in cost of materials and wages, and the grant was accordingly reduced to £6 10s., a further reduction being subsequently made to £6, and last March to £5. In order to encourage the speedy carrying out of this work, and at the same time to give the private contractor a fair chance, the Corporation contribution has been slightly more than half the average cost. Of the total conversions completed, the Corporation have only carried out 17 per cent. by direct labour, as the owners have preferred to take advantage of the grant. The result has been that although this scheme was only actually commenced a little over a year ago, over 6,000 conversions have been completed, leaving only 3,000 pails now outstanding in the borough, which we contemplate will be converted within the next few months. Over 1,000 conversions were completed during February.

Prior to commencing work on the scheme, a map was prepared showing the privies and pails in existence in the borough, and it was decided to serve notices in the districts on the outskirts of the borough first, working gradually in towards the centre. The effect of this method of procedure was that, as the scheme progressed, the area in which pail closets were still outstanding became more and more concentrated centrally and comparatively near the depot at which the contents of the pails was emptied ; consequently the number of men employed on the collection of pails could be reduced not only in proportion to the number of pail closets converted, but still further, in view of the considerably reduced area to be dealt with, and it was found when half the scheme had been completed that it was only necessary to employ three drivers and nine pail men, whereas previously nine drivers and twenty men were employed.

When the Corporation pay part of the cost of conversion it is subject to the work being carried out in compliance with a standard specification. Before beginning the work we specified that the height of the cistern was to be not less than 4 ft. 6 in. from the top of the closet seat. If these regulations had been adhered to it would have meant, in the majority of cases, lifting the roof of existing closet structures, and we found by experiments that almost as good a flush could be obtained with a reduced height of 3 ft. 6 in. above the seat. The specification was altered accordingly, which effected an average saving of £2 per conversion. Another important point is to see that the weight of the W.C. basin is not less than 40 lb. and that the whole work is carried out in compliance with the regulations of the Waterworks Committee. In the past, in the event of industrial disputes in towns where privies and pails exist, they were a grave menace to the public health, as they were left for a considerable time without being emptied pending the settlement of the dispute. As the pails primarily existed in working-class districts, it was the industrial classes themselves who ran this great danger, which will now be removed.

It may be suggested that the time is not opportune from a financial standpoint to carry out this important reform, but I consider that the time was never more ripe for doing this work, as the ratepayer, the taxpayer, the property-owner and the tenant will benefit. At the present time there is an exceptional amount of unemployment. The conversion scheme when completed will displace 30 men, but has provided most useful work for 100 men per week who would otherwise have been receiving doles, and it is better to pay wages for such useful work than doles for enforced idleness. The most important advantage to be gained is, however, from a health point of view, as every privy and pail closet constitutes a serious menace to the health of the people, the emptying is a constant source of pollution to the soil, and the affected soil is trodden into the houses by the people.

---

The Voluntary Hospitals Commission has offered a grant of £8,000 to the General Hospital, Birmingham, on condition that the beds closed from lack of funds be reopened. An undertaking has also been given that they shall not be closed again, since the number of waiting cases is heavy. This means that the beds at the General Hospital and at the Jaffray Hospital, Erdington, will be available so soon as the necessary staff has been collected. They will probably not be occupied before October.

